# A New Gaseous Disinfectant

**Published:** 1881-11

**Authors:** 


### A NEW GASEOUS DISINFECTANT.

M. Peyrusson has communicated to the Académie des Sciences the result of a series of experiments made with the fumes of nitrous ether. From these it has been demonstrated that they possess a most manifest disinfectant and anti-putrid action.

In addition to the trials made with it in the laboratory, it has been employed in the hospitals of Limoges. This agent has successfully purified the wards. It has also been used to preserve cadavers.

This ether offers another advantage: its odor is mild, agreeable, and entirely inoffensive.—*Monthly Review of Medicine and Surgery.*

